# Nursing care of a giant acoustic neuroma patient complicated with tonsillar herniation in the postoperative period: A CARE-compliant case report

**DOI:** 10.1097/MD.0000000000046871

**Published:** 2025-12-26

**Authors:** Yanfei Chen, Guanhua Hou

**Affiliations:** a Department of Nursing, The Fourth Affiliated Hospital of School of Medicine, and International School of Medicine, International Institutes of Medicine, Zhejiang University, Yiwu, China.

**Keywords:** giant acoustic neuroma, nursing care, postoperative period, tonsillar herniation

## Abstract

**Rationale:**

Giant Aacoustic neuroma resection has high risks of postoperative vital sign changes and consciousness disturbances. Brain edema or delayed hemorrhage may cause life-threatening tonsillar herniation, but systematic nursing reports are lacking for high-risk cases, especially giant acoustic neuroma patients with tonsillar herniation after partial resection. This study aimed to summarize comprehensive nursing strategies for such patients to provide clinical references for improving prognosis, with “Internet + extended care” as a unique post-discharge measure to promote recovery.

**Patient concerns:**

A 36-year-old male was admitted for half-month unsteady gait with no family history or comorbidities. Examinations showed persistent needle-like headache, right ear hearing loss, conjunctival congestion, horizontal nystagmus, right eye abduction limitation, incomplete eyelid closure, hoarseness and positive Romberg sign.

**Diagnoses:**

Preoperative clinical examination and enhanced cranial MRI confirmed a right cerebellopontine angle occupying lesion.

**Interventions:**

Nursing included a multidisciplinary team with feedforward control, early tonsillar herniation identification, refined eye care, whole-process swallowing assessment and rehabilitation, tidal-style psychological support. Post-discharge “Internet + extended care” (online health education, self-management monitoring, home services, psychological therapy) was used to improve motor, mental, cognitive and language functions.

**Outcomes:**

The patient regained consciousness 2 days after the second emergency surgery with glasgow coma scale score 4 + T + 6, muscle strength grade 5 and normal pupil size and light reflex. Functional oral intake scale grade was 3 on postoperative day 13, 5 on day 20 (nasogastric tube removed), and 6 2 days before discharge (full oral intake without aspiration). He was discharged ambulatorily on postoperative day 30 with no keratitis or corneal ulceration. Hamilton anxiety rating scale score decreased from 31 to 18, Hamilton depression rating scale from 25 to 16. One month after discharge, ADL score increased from 70 to 95 with no falls, and he reintegrated into family and social life and completed 3-month postoperative follow-up.

**Lessons:**

This study provides a guarantee for the comprehensive nursing of patients with tonsillar herniation after posterior fossa surgery and has important clinical reference value.

## 1. Introduction

Acoustic neuromas originate from the vestibular nerve sheath and account for approximately 8% of all intracranial tumors.^[1]^ Located at the cerebellopontine angle of the posterior fossa, giant acoustic neuromas can compress and displace the brainstem to form adhesions. Giant acoustic neuromas are deeply positioned and intimately associated with the surrounding structures, including the cerebellum, brainstem, blood vessels, and cranial nerves. Surgery is the primary therapeutic modality for the treatment of this disease. However, surgical resection of giant acoustic neuromas is technically challenging, with postoperative complications, such as hearing loss, tinnitus, headache, facial numbness, ataxia, vertigo, and other comprehensive symptoms.^[2]^ Direct or indirect injury to brainstem function during surgery may lead to postoperative alterations in respiration, heart rate, blood pressure, and consciousness.^[3]^

In March 2023, our department admitted a patient with giant acoustic neuroma who underwent acoustic neuroma resection. The surgical procedure was uneventful, and the patient achieved a Glasgow coma scale (GCS) score of 15. However, the patient developed complications such as dyspnea and brain herniation. Through the modification of conventional nursing protocols, early identification of brain herniation, and implementation of comprehensive interventions, a favorable prognosis was achieved. Despite postoperative facial paralysis, swallowing dysfunction, and significant negative emotions, the patient was successfully treated and reintegrated into family and social life through active nursing strategies and psychological support. This case report is organized as follows.

## 2. Case description

**Figure 1.**
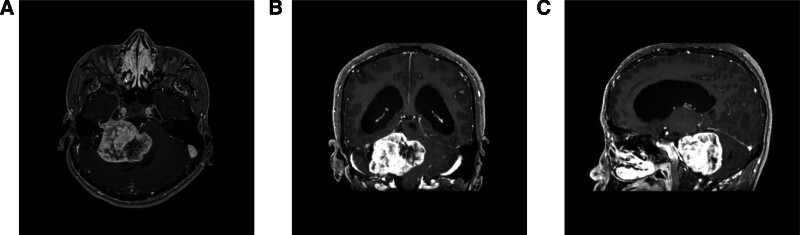
Preoperative CE-MRI. (A–C) Axial, coronal, and sagittal views of the tumor, respectively. CE-MRI = contrast-enhanced magnetic resonance imaging.

A 36-year-old male patient was admitted to the hospital in March 2023 because of an unsteady gait for half a month. This patient has no family history or comorbidities. Enhanced cranial MRI revealed an occupying lesion at the right cerebellopontine angle (Fig. 1). Physical examination revealed bilateral pupils equal in size and reactive to light, and grade 5 muscle strength in all 4 limbs, unsteady gait, persistent needle-like headache without relief, and hearing loss in the right ear. The patient presented with conjunctival congestion and edema, horizontal nystagmus, right eye abduction limitation, incomplete eyelid closure, hoarseness, and a positive Romberg sign.

On March 7, 2023, the patient underwent the right retrosigmoid approach for acoustic neuroma resection and external ventricular drainage. The procedure was uneventful, and the patient remained conscious postoperatively with a GCS score of 15 and bilateral pupils of 3 mm in diameter with brisk light reflexes. Postoperative management included mannitol treatment to reduce the intracranial pressure and nutritional support.

**Figure 2.**
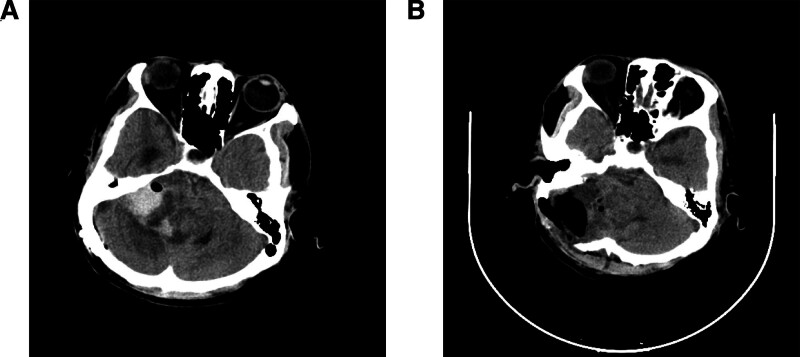
(A) CT showed cerebellar swelling and hematoma on March 10. (B) Postoperative imaging showed the hematoma was cleared. CT = computed tomography.

At 09:25 on March 10, the patient developed a posterior tongue fall, respiratory rate of 25 to 28 per minute, and heart rate fluctuating between 126 and 141 beats per minute. An immediate tracheostomy was performed. At 18:09, the patient’s GCS score was 3 + T + 5 and the pupils were 5/4 mm with a delayed light reflex. CT revealed cerebellar swelling and hematoma (Fig. 2A). Emergency intracranial hematoma evacuation and partial cerebellectomy were then performed. Postoperative imaging showed that the hematoma had been cleared (Fig. 2B).

**Figure 3.**
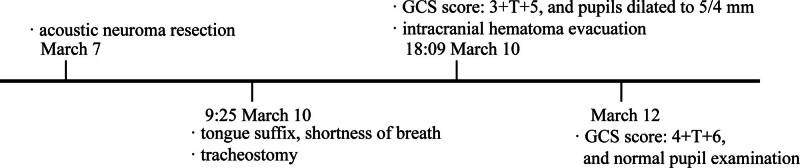
Diagnosis and treatment process.

Two days after surgery, the patient regained consciousness with a GCS score of 4 + T + 6, grade 5 of muscle strength, and normal pupil examination. Thirty days postoperatively, the patient was discharged ambulatory and successfully reintegrated into family and social life. Timeline of diagnosis and treatment process see in Figure 3.

Informed consent was obtained from the patient. This study was supported by the Ethics Committee of the Fourth Affiliated Hospital of Zhejiang University School of Medicine.

## 3. Discussion

Giant acoustic neuromas arising from the vestibular nerve sheath constitute a subset of intracranial tumors, with a predilection for the cerebellopontine angle in the posterior fossa. Surgical resection remains the primary therapeutic modality and is accompanied by a spectrum of postoperative complications including cranial nerve deficits, cerebellar ataxia, intracranial hypertension, and vital sign instability. Among these, tonsillar herniation is the most time-critical complication, because early recognition and intervention are pivotal for improving patient outcomes. We summarized the nursing care experience of giant acoustic neuroma in a patient with tonsillar herniation in the perioperative period.

### 3.1. Establishment of feedforward-controlled multidisciplinary team

A multidisciplinary team (MDT) comprised of neurosurgeons, intensivists, anesthesiologists, dietitians, rehabilitation physicians, neurosurgical nurses, and intensive care nurses. The MDT focused on a comprehensive assessment of surgical risks and potential postoperative complications. The nurse oversaw the patient’s underlying medical condition and organized rehearsals for emergency response protocols targeting high-risk postoperative complications. Emergency management of delayed hemorrhage, intracranial hematoma, or postoperative brain herniation requiring urgent surgery. The nurse immediately coordinated preoperative preparation and transport arrangements, including activating the surgical elevator and transferring the patient to the operating room. Tracheostomy tubes, tapes, and kits were prepared at the bedside according to the patient’s anatomical characteristics for potential bedside tracheostomy. During cardiopulmonary arrest, nurses and physicians initiate immediate cardiopulmonary resuscitation (CPR). The ward secretary or on-duty nurse activates the hospital emergency response team with anesthesiologists performing urgent bedside endotracheal intubation. Subsequent decisions regarding intensive care unit transfer or emergency surgery were confirmed by the attending physician.

### 3.2. Early identification and emergency management of tonsillar herniation

Based on the preoperative MDT assessment, the patient was at high risk of vital sign instability due to brainstem involvement, with tonsillar herniation being the primary concern. This condition typically manifests initially as vital sign derangements that may progress to sudden cardiac arrest in severe cases. Within the first 6 hours, vital signs were monitored every 15 minutes versus standard 30-minute intervals, with special attention to oxygen saturation and respiratory rate during postoperative monitoring intensification. The tracheostomy kit was maintained at the bedside. Vital signs were recorded every 30 minutes, and neurological assessments, including GCS score, pupil response, and motor function, were conducted hourly. At 09:25 on postoperative day 3, the patient developed posterior tongue fall, tachypnea, tachycardia, and SpO2 of 92% via a Venturi mask with 40% oxygen. The GCS score was 4 + 4 + 6, with normal pupil response and muscle strength. CT revealed exacerbated local brain edema in the operative field. Anticipating respiratory deterioration, the team immediately performed a tracheostomy and initiated mechanical ventilation, resulting in improved vital signs. Therapeutic adjustments included intravenous methylprednisolone (40 mg) and mannitol (250 ml every 6 hours). At 18:09, the patient’s GCS score decreased to 3 + T + 5, with pupils dilated to 5/4 mm and a blunting light reflex. Computed tomography (CT) revealed cerebellar swelling and hematoma, which triggered an interdisciplinary critical patient transport protocol. The nurse initiated an urgent surgical plan, and the patient underwent emergency intracranial hematoma evacuation, craniotomy for decompression, and partial cerebellectomy. Two days after the operation, consciousness recovered, and vital signs stabilized.

### 3.3. Refined ocular care management

The patient presented with limited right eye abduction, ptosis, and conjunctival chemosis. Postoperatively, symptoms of facial nerve palsy emerged, including hypesthesia, incomplete eyelid closure, exposure keratitis, and corneal ulceration. Educate the patient on eye hygiene to avoid rubbing eyes and provide an eye shield for nighttime use during nursing care. Artificial tears 4 times daily, and levofloxacin ophthalmic ointment was administered once every night per physician. Once conscious, the patient underwent bedside facial acupuncture therapy performed daily by a rehabilitation specialist. The prescribed facial muscle exercise regimen focusing on the levator palpebrae superioris^[4]^ includes maximal elevating of the eyebrows for 5 seconds. The brow position was maintained for 5 seconds during squinting. These 3 sets were repeated twice a day. Oral mecobalamin (0.5 mg) was administered thrice daily to support nerve regeneration. At discharge, the patient showed no signs of keratitis or ulceration and had mastered the facial exercise protocol.

### 3.4. Comprehensive swallowing function screening and rehabilitation

Upon admission, the nurse immediately conducted a swallowing dysfunction assessment, which revealed moderate choking during the Kubota Drinking Test. A bedside video-vesicular swallowing test (V-VST) performed by a rehabilitation physician showed a Functional Oral Intake Scale (FOIS) grade of 2, indicating recommendations for enteral nutrition with supervised minimal oral intake. A nasogastric tube was inserted, and 1500 ml/day of enteral nutrition formula was administered. The charge nurse and rehabilitation physician provided bedside guidance for the seated intake of pureed foods, emphasizing the strict avoidance of unsupervised self-feeding. Bedside swallowing exercises were initiated preoperatively and postoperatively with stabilization of the condition. Swallowing function training included ice and dry swallowing exercises. Vocal cord closure training via breath-holding maneuvers and laryngeal elevation exercise. Feeding training was performed by instructing the patient to assume a 30° supine position with the head flexed forward. Begans with easily swallowed pureed foods progress sequentially to thin liquids, semisolids, and solids. Emphasizing optimal bolus size and swallowing techniques. Electrical stimulation therapy was administered at a tolerable intensity to induce muscle contraction and guide active swallowing during stimulation. The patient’s condition advanced to FOIS grade 3 on postoperative day 13 and grade 5 on postoperative day 20, necessitating nasogastric tube removal. Two days before discharge, the patient achieved FOIS grade 6, demonstrating a full oral intake capacity. No complications such as aspiration occurred during exercise

### 3.5. Tidal model of metal health nursing

Following the deterioration of the patient’s condition, severe insecurity emerged, characterized by limited communication with family members, inability to speak because of tracheostomy, and pronounced negative emotions. To address these issues, tidal model of mental health nursing was implemented, providing support across 3 domains: “self,” “world,” and “others.”^[5]^ Each domain targets specific psychological needs, including emotional and safety requirements, need for empathetic listening, and establishment of personal boundaries or expressions of individual preferences. “Self” domain: preoperatively, the patient exhibited concerns about surgical prognosis and regret for delayed medical attention, with Hamilton Anxiety Rating Scale (HARS) score of 20 and Hamilton Depression Rating Scale (HDRS) score of 16. The nurse provided targeted health education on acoustic neuroma, including potential complications, to alleviate the anxiety associated with the unknown. Real-world domain: After regaining consciousness, the patient became acutely aware of recurrent critical events and experienced a sense of impending doom, fatigue, and heightened distress owing to muteness. The HARS and HDRS scores had increased to 31 and 25, respectively. Daily psychological support focuses on 3 aspects: self-management strategies, the narrative processing of personal experiences, and satisfaction with emotional needs. Given the patient’s inability to speak after tracheostomy, the nurse encouraged communication via mobile phone typing, prompting the expression of negative emotions, and facilitating rapid interventions to improve subjective well-being. “Others” domain: Success stories of patients who recovered well after acoustic neuroma surgery and tracheostomy decannulation were shared. Additionally, visits from family members, friends, and relatives of patients with successful decannulation were arranged to offer emotional support and hope for recovery. Upon discharge, the patient’s HARS and HDRS scores decreased to 18 and 16, respectively, indicating a significant improvement in psychological state.

### 3.6. Internet + extended care

Owing to incomplete tumor resection, the patient required enhanced follow-up beyond the standard 2-week outpatient visit. Fixed telephone follow-ups were scheduled at 1 week, 1 month, 2 months, and 3 months postoperatively, complemented by Internet + services via the “Zhejiang Nursing” platform. The improvement of motor function, promotion of mental health, enhancement of cognitive level, and amelioration of language function were achieved through online health education, supervision of self-management, “Internet +” home-based services, and online psychotherapy. We focused on rehabilitation and safety care a month postoperatively, including guidance on self-monitoring for neurological changes and fall prevention, and supervised home-based rehabilitation exercises to promote independence. The patient’s ADL score improved from 70 at discharge to 95 within 1 month, with no reported falls. to 1 to 3 months should focus on facilitating social reintegration and coordinated follow-up care, including encouraging a gradual return to daily activities and community participation. Assisted in scheduling and completing the 3-month postoperative imaging and clinical evaluation.

## 4. Limitations

This study has several limitations that need to be acknowledged. Firstly, it is a single-case retrospective report without a control group, which means the described clinical situation and nursing outcomes are specific to this individual patient. Thus, the generalizability of the summarized nursing strategies to a broader population of patients with giant acoustic neuroma complicated by tonsillar herniation is limited. Secondly, the study did not systematically evaluate intervention adherence and patient tolerability during the nursing process, which may affect the objectivity of the effectiveness assessment of the nursing measures. Thirdly, due to the retrospective nature, some potential adverse events or unanticipated situations during the perioperative period may not have been fully recorded, leading to possible information bias. Future studies should consider conducting prospective controlled trials with a larger sample size to verify the effectiveness of the proposed nursing strategies, and extend the follow-up period to obtain more comprehensive long-term outcome data.

## 5. Conclusion

Acoustic neuroma resection is a complicated neurosurgical procedure, particularly in cases of giant tumors that invade and compress brainstem structures, posing a high risk for complete resection.^[6]^ The perioperative period of this patient was complicated by dysphagia, facial paralysis, postoperative brain herniation, 2 surgical interventions, and tracheostomy, which led to multiple postoperative morbidities. Concomitantly, the patient and family exhibited pronounced negative emotions, including anxiety, fear, and sadness. Comprehensive nursing interventions were key to favorable outcomes: feedforward-controlled MDT for risk assessment, early complication management, specialized care for organ dysfunctions, and tidal-model psychological support. Notably, “Internet + extended care” was irreplaceable for long-term rehabilitation – via online education, remote monitoring, and counseling, the patient’s ADL score rose from 70 to 95, with no falls, and successfully reintegrated into life. This study shows a multi-dimensional nursing approach improves prognosis, providing clinical references for similar high-risk patients.

## Author contributions

**Writing** – **original draft:** Yanfei Chen.

**Writing** – **review & editing:** Guanhua Hou.
